# Regulation of histone H2A.Z expression is mediated by sirtuin 1 in prostate cancer

**DOI:** 10.18632/oncotarget.1237

**Published:** 2013-08-29

**Authors:** Tiago Baptista, Inês Graça, Elsa J. Sousa, Ana I. Oliveira, Natália R. Costa, Pedro Costa-Pinheiro, Francisco Amado, Rui Henrique, Carmen Jerónimo

**Affiliations:** ^1^ Cancer Epigenetics Group, Research Center, Portuguese Oncology Institute – Porto, Portugal; ^2^ Department of Genetics, Portuguese Oncology Institute – Porto, Portugal; ^3^ Department of Pathology, Portuguese Oncology Institute – Porto, Portugal; ^4^ School of Allied Health Sciences ESTSP, Polytechnic of Porto; ^5^ Department of Chemistry, Universidade de Aveiro, Campus Universitário de Santiago, Aveiro, Portugal; ^6^ Department of Pathology and Molecular Immunology, Institute of Biomedical Sciences Abel Salazar, University of Porto, Portugal

**Keywords:** Prostate cancer, H2A.Z, sirtuin 1, epigenetic drugs

## Abstract

Histone variants seem to play a major role in gene expression regulation. In prostate cancer, H2A.Z and its acetylated form are implicated in oncogenes' upregulation. *SIRT1*, which may act either as tumor suppressor or oncogene, reduces H2A.Z levels in cardiomyocytes, via proteasome-mediated degradation, and this mechanism might be impaired in prostate cancer cells due to sirtuin 1 downregulation. Thus, we aimed to characterize the mechanisms underlying H2A.Z and *SIRT1* deregulation in prostate carcinogenesis and how they interact.

We found that *H2AFZ* and *SIRT1* were up- and downregulated, respectively, at transcript level in primary prostate cancer and high-grade prostatic intraepithelial neoplasia compared to normal prostatic tissues. Induced *SIRT1* overexpression in prostate cancer cell lines resulted in almost complete absence of H2A.Z. Inhibition of mTOR had a modest effect on H2A.Z levels, but proteasome inhibition prevented the marked reduction of H2A.Z due to sirtuin 1 overexpression. Prostate cancer cells exposed to epigenetic modifying drugs trichostatin A, alone or combined with 5-aza-2'-deoxycytidine, increased *H2AFZ* transcript, although with a concomitant decrease in protein levels. Conversely, *SIRT1* transcript and protein levels increased after exposure. ChIP revealed an increase of activation marks within the TSS region for both genes. Remarkably, inhibition of sirtuin 1 with nicotinamide, increased H2A.Z levels, whereas activation of sirtuin 1 by resveratrol led to an abrupt decrease in H2A.Z. Finally, protein-ligation assay showed that exposure to epigenetic modifying drugs fostered the interaction between sirtuin 1 and H2A.Z.

We concluded that sirtuin 1 and H2A.Z deregulation in prostate cancer are reciprocally related. Epigenetic mechanisms, mostly histone post-translational modifications, are likely involved and impair sirtuin 1-mediated downregulation of H2A.Z via proteasome-mediated degradation. Epigenetic modifying drugs in conjunction with enzymatic modulators are able to restore the normal functions of sirtuin 1 and might constitute relevant tools for targeted therapy of prostate cancer patients.

## INTRODUCTION

Prostate cancer (PCa) is the second most common malignancy diagnosed in men worldwide, being one of the major causes of cancer-related morbidity and mortality[1]. Although mostly symptomatic only in advanced stages, PCa is characterized by early disruption of both genetic[2] and epigenetic[3] mechanisms, progressively becoming more severe throughout disease progression.

Alterations in histone variants are the least understood among epigenetic mechanisms deregulated in cancer. Histone variant H2A.Z, encoded by *H2AFZ*, whose insertion into chromatin is tightly regulated by complexes such as SRCAP, p400/Tip60 and TIP48/49 [4], plays a major role in critical biological processes, such as chromosome segregation [5], cell cycle progression [6] and maintenance of heterochromatin/euchromatin status [7]. Nevertheless, H2A.Z has been consistently associated with neoplastic transformation: *H2AF*Z overexpression has been reported in sporadic microsatellite unstable colorectal carcinoma [8], breast carcinoma [9,10] and several undifferentiated cancers [11]. At a molecular level, this histone variant is responsible for overexpression of several proto-oncogenes, including *C-MYC* [9,12]. In PCa, however, the role of H2A.Z remains elusive [13]. Increased expression of *H2AFZ* was found in a castration-resistant xenograph model of PCa, suggesting that high levels of H2A.Z might be predictive to progression for androgen-independent disease [14]. Conversely, it has been claimed that acetylated H2A.Z (acH2A.Z), and not H2A.Z itself, was enriched (oncogenes) or lost (tumor suppressor genes, TSG) at the transcription start-site (TSS) of nucleosomes during carcinogenesis, suggesting that acH2A.Z contributes for gene expression deregulation in PCa [15].

It has been previously reported that sirtuin 1, a member of class III histone deacetylases (HDACs), negatively regulates H2A.Z levels in cardiomyocytes, through targeting of this histone variant to degradation via an ubiquitin/proteasome pathway [16]. However, the role of sirtuin 1 in carcinogenesis remains unclear [17, 18]. Indeed, this HDAC is overexpressed in some cancers [19, 20], but downregulated in others [21], supporting its role either as an oncogene or a TSG. In PCa, both *SIRT1* over- and underexpression have been reported [21, 22]. Nevertheless, there is accumulating evidence that sirtuin 1 mainly acts as a tumor suppressor protein [23-25], due to its ability to promote the activity of TSC2, a repressor of mTOR [26].

In this study, we aimed to uncover the putative regulatory role of sirtuin 1 in H2A.Z expression during prostate carcinogenesis and determine whether it might constitute a relevant therapeutic target for PCa.

## RESULTS

### SIRT1 and H2AFZ are deregulated in PCa

*H2AFZ* and *SIRT1* transcript levels were assessed in primary PCa, as well as in high-grade prostatic intraepithelial neoplasia (PIN) and morphologically normal prostate tissue (NPT). Relevant clinical and histopathological data are depicted in Table 1. No statistically significant differences were found for age between patients and controls (NPT).

**Table 1:** Clinical and histopathological features of patient populations

Clinicopathological Features	NPT	PIN	PCa
Patients, n Median age, median (range) PSA (ng/mL), median (range)	10 63 (45 - 79) n.a.	10 63 (51 - 73) n.a.	57 66 (51 - 74) 9.05 (2.66 - 35.50)
Pathological Stage, n (%)			
pT2	n.a.	n.a.	23 (40)
pT3	n.a.	n.a.	34 (60)
Gleason Score, n (%)			
< 7	n.a.	n.a.	12 (21)
= 7	n.a.	n.a.	39 (68)
> 7	n.a.	n.a.	6 (11)

NPT - morphologically normal prostate tissue, PIN - prostatic intraepithelial neoplasia, PCa - prostate carcinoma, n.a. - not applicable.

**Figure 1:**
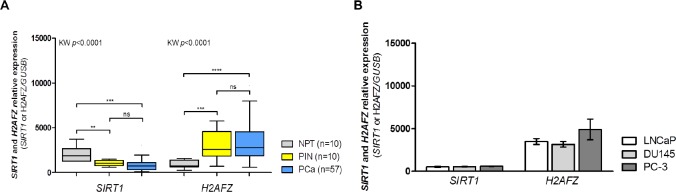
Transcriptional status of *SIRT1* and *H2AFZ* in clinical samples (normal prostate tissues – NPT –, prostatic intraepithelial neoplasia – PIN – and prostate carcinoma - PCa) and PCa cell lines (LNCaP, DU145 and PC-3) (A) *SIRT1* downregulation and *H2AFZ* overexpression are early events in prostatic carcinogenesis (group analysis with Kruskal-Wallis test followed by a pairwise Mann-Whitney *U* test, **p<0.01, ***p<0.001 and ****p<0.0001); (B) *SIRT1* and *H2AFZ* gene expression in PCa cell lines follow the tendency observed for clinical samples of prostatic malignancy (mean ± SD, n=3).

Statistically significant differences were observed in *SIRT1* and *H2AFZ* transcript levels among the three analyzed groups. Both PIN lesions and PCa showed downregulation of *SIRT1* with concomitant overexpression of *H2AFZ*, compared to NPT (Fig. 1A). No statistically significant differences were disclosed for *SIRT1* and *H2AFZ* expression levels between PIN and PCa samples, and no associations were found with clinicopathological variables in PCa patients.

Expression profiling of PCa cell lines LNCaP, DU145, and PC-3 revealed that *SIRT1* and *H2AFZ* mRNA levels were within the same range as that observed in primary PCa tissue samples (Fig. 1B).

### Overexpression of SIRT1 decreases levels of H2A.Z independently of mTOR inhibition

**Figure 2:**
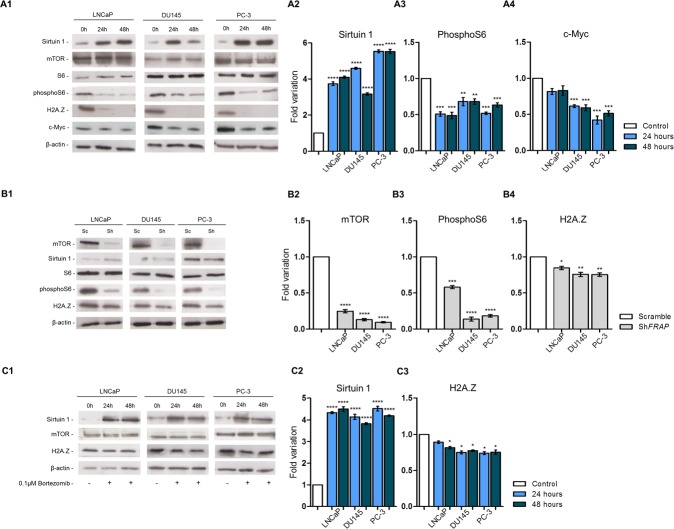
Protein profile of three PCa cell lines - LNCaP, DU145 and PC-3 – after (A1) *SIRT1* overexpression, (B1) mTOR silencing and (C1) *SIRT1* overexpression and exposure to bortezomib After *SIRT1* overexpression levels of (A2) sirtuin 1 increased, with concomitant decrease in (A3) pS6, (A4) c-Myc and H2A.Z. *FRAP* silencing induced a decrease in (B2) mTOR, (B3) pS6 and (B4) H2A.Z. When *SIRT1* overexpressing cells were exposed to bortezomib, the increased levels of (C2) sirtuin 1 caused a significant decrease in H2A.Z levels (C3). However, this decrease was much less impressive than previously observed for *SIRT1* upregulation without proteasome inhibition. Results were normalized to β-actin and are presented as fold variation in comparison to control (mean±SD, n=3). Dunnet's test: *p<0.05, **p<0.01, ***p<0.001, ****p<0.0001.

To investigate the role of *sirtuin 1* in the modulation of H2A.Z expression, *SIRT1* overexpression was induced in LNCaP, DU145 and PC-3 cell lines [validation of successful transduction was assessed by qRT-PCR (Supplementary Fig. 1A) and Western blot (Fig. 2 A1 and 2 A2)]. Following induction of *SIRT1* expression, a significant reduction of phosphorylated ribosomal protein S6 (phosphoS6, an effector of mTOR pathway) was found, although mTOR and ribosomal protein S6 (S6) remained unchanged (Figs. 2 A1 and 2 A3). In addition, H2A.Z protein suffered an impressive reduction to nearly undetectable levels (Fig. 2 A1), in parallel with a significant reduction of its target c-Myc, in DU145 and PC-3 cells (Fig. 2 A4).

In order to assess whether decreased H2A.Z levels were due to mTOR pathway inhibition by sirtuin 1, the selected PCa cell lines were stably silenced for *FRAP*, the gene encoding mTOR protein, and silencing validation was performed by qRT-PCR (Supplementary Fig. 1B) and Western blot (Fig. 2 B1 and 2 B2). As expected, mTOR pathway inhibition decreased phosphoS6 levels (Fig. 2 B3). Furthermore, a statistically significant, but modest reduction in H2A.Z levels was observed following *FRAP* silencing (*Fig. 2 B4*).

### Pharmacological inhibition of proteasome impairs H2A.Z degradation caused by sirtuin 1 induced overexpression

Because sirtuin 1 might induce H2A.Z degradation through the proteasome, we evaluated whether H2A.Z decrease might be due to that mechanism. Hence, the three PCa cell lines were transiently transfected for *SIRT1* and exposed to 0.1μM bortezomib, a selective inhibitor of proteasomal activity (Fig. 2 C1). Although *SIRT1* overexpression (Supplementary Fig. 1C and Fig. 2 C2) and concomitant proteasome inhibition were associated with a decrease in H2A.Z levels (Fig. 2 C3), the extent of this decrease was considerably less impressive when compared to *SIRT1* overexpression without proteasome inhibition.

### Trichostatin A (TSA) leads to increased expression of both H2AFZ and SIRT1 through regulation of histone marks

**Figure 3:**
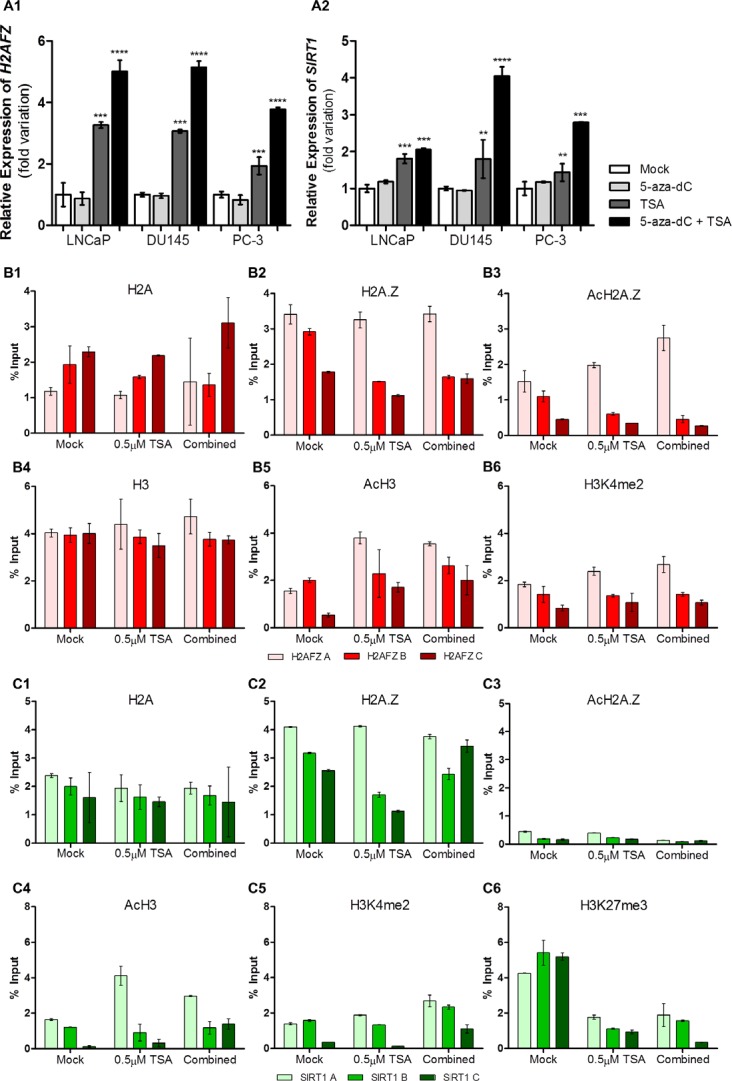
Epigenetic modulating drugs positively regulate *H2AFZ* and *SIRT1* transcription Transcript levels of (A1) *H2AFZ* and (A2) *SIRT1* in three distinct PCa cell lines after exposure to 5-aza-dC and/or TSA. The results are presented as fold variation in comparison to the experimental control (mean±SD, n=3). Dunnet's test: **p<0.01, ***p<0.001, ****p<0.0001. ChIP assay results in LNCaP cell line regarding (B1) H2A, (B2) H2A.Z, (B3) AcH2A.Z, (B4) H3, (B5) AcH3 and (B6) H3K4me2 histones and histones marks across *H2AFZ* promoter and regarding (C1) H2A, (C2) H2A.Z, (C3) AcH2A.Z, (C4) AcH3, (C5) H3K4me2 and (C6) histones and histones marks along *SIRT1* promoter. Results are normalized with the input of total sonicated chromatin (mean ± SD).

To ascertain whether epigenetic modulating drugs were able to regulate *H2AFZ* and *SIRT1* transcriptional status, PCa cell lines were exposed to 5-aza-2'-depxycytidine (5-aza-dC) and/or TSA. TSA, alone or combined with 5-aza-dC, led to an increase in mRNA levels for both genes, whereas 5-aza-dC alone was not able to significantly alter their expression (Fig. 3 A1 and 3 A2). Hence, the effect of 5-aza-dC alone was not further explored.

Subsequently, chromatin immunoprecipitation (ChIP) was performed in the three selected PCa cell lines after exposure to TSA (Fig. 3B/3C and Supplementary Figs. 2, 3 and 4). For LNCaP cells, concerning *H2AFZ*, no relevant differences were found for H2A and H2A.Z levels within the promoter, after treatment. However, differences in distribution were depicted as H2A tended to accumulate away from the TSS (Fig. 3 B1), whereas H2A.Z was enriched nearby the TSS (Fig. 3 B2), particularly its acetylated form, and after exposure to TSA (Fig. 3 B3). Concurrently, after drug exposure, H3 levels remained stable (Fig. 3 B4) whereas the distribution of the activating marks acH3 and H3K4me2 was increased closer to the TSS (Fig. 3 B5 and 3 B6). Nevertheless, no variations were observed for the repressive mark H3K27me3 along the full extent of the *H2AFZ* promoter (Supplementary Fig. 2). Remarkably, the same results were observed for DU145 and PC-3, except for AcH2A.Z in PC-3, which was not increased after treatment with both drugs (Supplementary Fig. 3 and 4).

Likewise, in LNCaP, although H2A.Z levels were higher than those of H2A, drug exposure did not alter H2A and H2A.Z levels across *SIRT1* promoter (Fig. 3 C1 and 3 C2), and both were particularly enriched nearby the TSS. Nonetheless, acH2A.Z levels remained very low and dispersed along the *SIRT1* promoter (Fig. 3 C3). Within this same promoter, alterations in H3 levels were found in LNCaP (Supplementary Fig. 2). Additionally, an increase in histone activating marks was demonstrated in nucleosomes close to TSS (Fig. 3 C4 and 3 C5) whereas a decrease in the H3K27me3 repressive mark was found after epigenetic drugs exposure (Fig. 3 C6). Concurrently, the same observations were found in DU145 and PC-3, although levels of H3 remained the same for both cell lines (Supplementary Figs. 3 and 4).

### Epigenetic modulating drugs reduce protein levels of H2A.Z with concomitant increase of sirtuin 1

**Figure 4:**
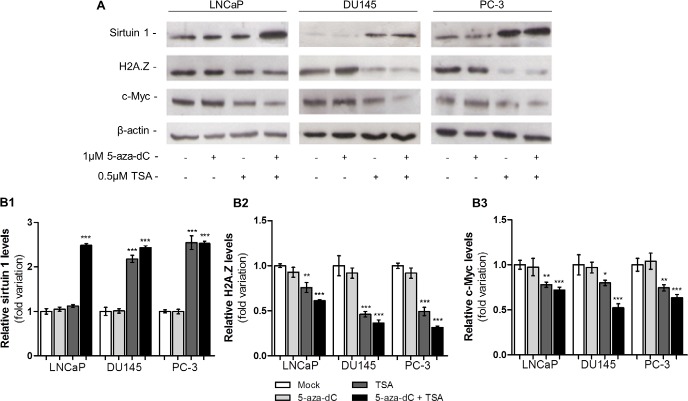
Epigenetic modulating drugs have the capacity to upregulate protein levels of sirtuin 1 and decrease levels of H2A.Z and c-Myc (A) Western blot analysis of sirtuin 1, H2A.Z and c-Myc of three PCa cell lines exposed to 5-aza-dC and/or TSA. Optical densities of visible bands for (B1) sirtuin 1, (B2) H2A.Z and (B3) c-Myc following treatment with 5-aza-dC and/or TSA. Intensity of the bands was normalized to the loading control β-actin and presented as folding variation in comparison to the untreated control (mean ± SD, n=3). Dunnet's test: *p<0.05, **p<0.01, ***p<0.001.

Because *SIRT1* and *H2AFZ* transcript levels increased after exposure to TSA alone or combined with 5-aza-dC, *H2AFZ* and *SIRT1* protein expression was further assessed (Fig. 4A). Intriguingly, whereas sirtuin 1 levels significantly increased after TSA exposure, alone or in combination with 5-aza-dC (Fig. 4 B1), the opposite trend was observed for H2AZ levels (Fig. 4 B2), as well as for c-Myc levels (Fig. 4 B3).

### Pharmacological inhibition or activation of sirtuin 1 directly affects regulation of H2A.Z protein levels

**Figure 5:**
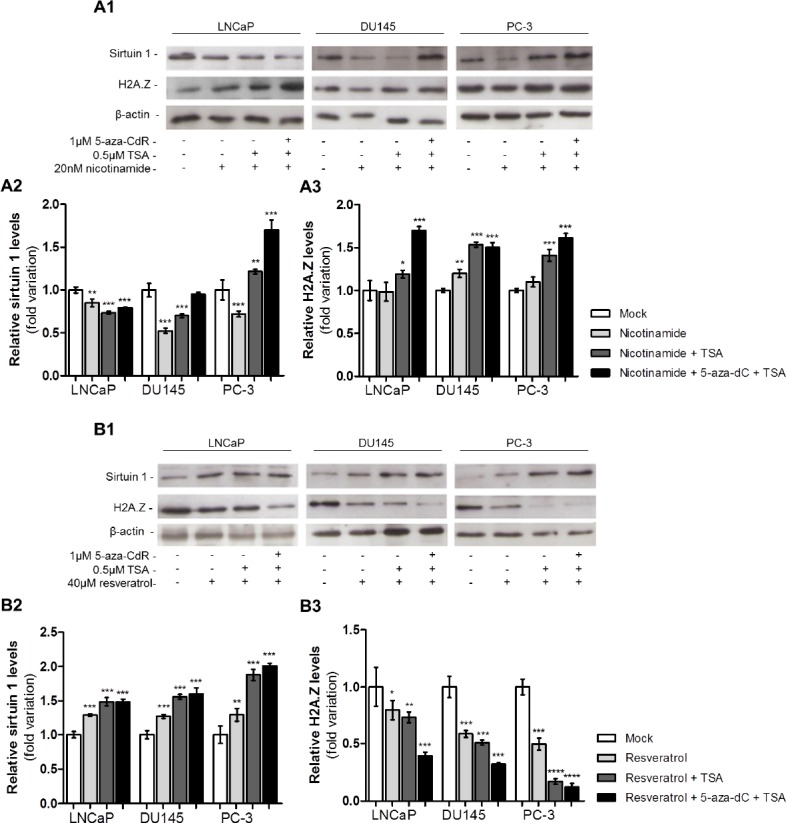
Pharmacological inhibition or activation of sirtuin 1 promotes H2A.Z up- or downregulation (A1) Western blot analysis of sirtuin 1 and H2A.Z of three PCa cell lines exposed to nicotinamide, alone or combined with 5-aza-dC and/or TSA. Optical densities of visible bands for (A2) sirtuin 1 and (A3) H2A.Z following drug exposure. (B1) Western blot analysis of sirtuin 1 and H2A.Z of three PCa cell lines exposed to resveratrol, alone or combined with 5-aza-dC and/or TSA. Optical densities of visible bands for (B2) sirtuin 1 and (B3) H2A.Z following drug exposure. Intensity of bands was normalized to the loading control β-actin and presented as folding variation in comparison to the untreated control (mean±SD, n=3). Dunnet's test: *p<0.05, **p<0.01, ***p<0.001.

The effect of sirtuin 1 pharmacological inhibition or activation on H2A.Z protein levels was assessed through exposure of PCa cell lines to the modulators of sirtuin 1 enzymatic activity nicotinamide (inhibitor) or resveratrol (activator). The same experiment was performed in combination with exposure to epigenetic modulating drugs (Figure 5).

Exposure to nicotinamide alone was associated with a decrease in sirtuin 1. Whereas in LNCaP cells, sirtuin 1 levels decreased after exposure to nicotinamide combined with both epigenetic drugs, in DU145 cells the same was observed only for nicotinamide combined with TSA. On the contrary, in PC-3 cells exposed to nicotinamide combined with TSA or 5-aza-dC plus TSA, an increase in sirtuin 1 levels was apparent (Fig. 5 A1 and 5 A2). An increase in H2A.Z protein levels after exposure to nicotinamide alone was observed only in DU145. Nonetheless, an increase in H2A.Z protein levels was observed in all cell lines when nicotinamide was combined with TSA or with both epigenetic drugs (Fig. 5 A1 and 5 A3).

In PCa cell lines exposed to resveratrol, a statistically significantly increase in sirtuin 1 protein levels was verified. This trend was more striking when the sirtuin 1 activator was used in combination with 5-aza-dC and TSA (Fig. 5 B1 and 5 B2). The opposite effect was observed for H2A.Z levels: cell lines exposed to resveratrol showed a decrease in H2A.Z levels. Interestingly, the lowest H2A.Z levels were found in PCa cell lines treated with resveratrol in combination with 5-aza-dC and TSA (Fig. 5 B1 and 5 B3).

**Figure 6:**
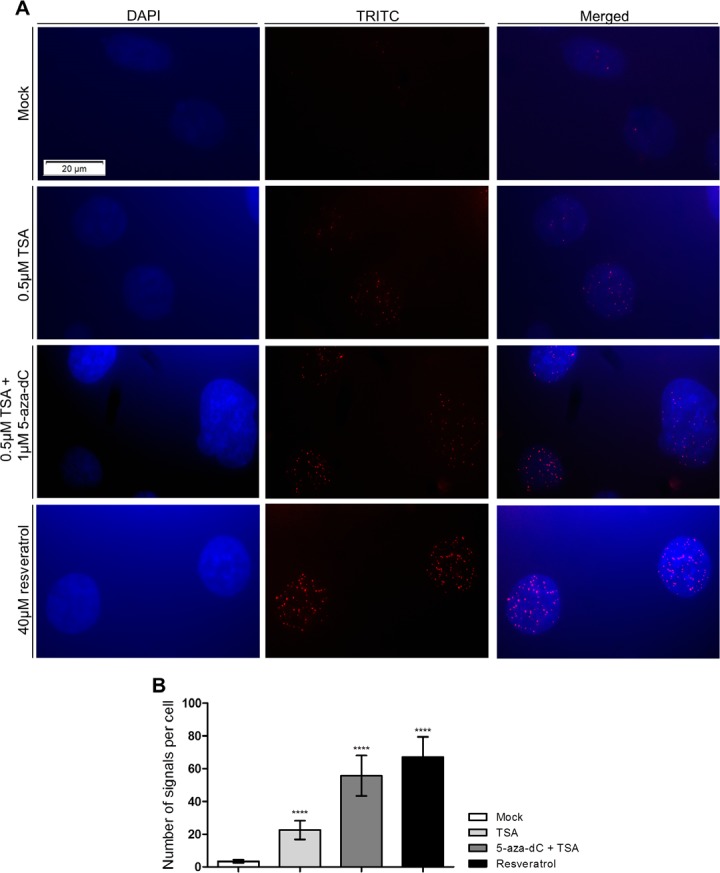
PCa cells exposure to epigenetic modulating drugs and resveratrol promotes the physical and direct interaction between sirtuin 1 and H2A.Z (A) PLA assay for PC-3 after exposure to 0.5μM TSA, alone or combined with 1μM 5-aza-dC, and 40μM resveratrol (40x magnification). (B) Analysis of PLA results represented as number of signals per cell (mean±SD, n=50). Dunnet's test: ****p<0.0001.

Finally, in PC-3 cell line exposed to epigenetic modulating drugs, a statistically significant increase in the interaction between sirtuin 1 and H2A.Z was demonstrated using PLA, and the same result was observed following exposure to resveratrol alone (Figs. 6A, 6B). Additionally, when cells were exposed to nicotinamide, a total absence of signals was depicted (data not shown).

## DISCUSSION

Histone variants seem to play a major role in gene expression regulation. Specifically, H2A.Z and acH2A.Z have been shown to be deregulated in some tumors, causing aberrant expression of several oncogenes [9, 15, 27, 28]. Moreover, sirtuin 1, a protein that may acts either as tumor suppressor or oncoprotein, has been previously shown to reduce H2A.Z levels in cardiomyocytes, via a cascade of events that ultimately leads to proteasome-mediated histone degradation [16]. Thus, we aimed to characterize the role of H2A.Z deregulation in prostate carcinogenesis and determine how sirtuin 1 might be involved in this process. After confirmation of deregulation of *SIRT1* and *H2AFZ* expression in primary PCa tissues, we found that, in PCa cell lines, induction of *SIRT1* expression led to a dramatic decrease of H2A.Z levels, mostly through proteasome-mediated degradation. Furthermore, exposure to epigenetic modulating drugs, mainly TSA, was able to reverse the concomitant deregulation of both genes in PCa at transcript and protein level. In addition to histone modification, direct interaction of sirtuin 1 and H2A.Z at protein level is also involved in sirtuin 1 regulation of *H2AFZ* expression.

This is the first report of altered *H2AFZ* expression in localized primary PCa, as well as in its putative precursor lesion, PIN. Interestingly, there is a previous publication on a trend for increased H2A.Z expression in tumors from patients submitted to androgen deprivation therapy, suggestive of a subset of castration-resistant tumors over-expressing this histone variant [14]. Concurrently, *SIRT1* decreased expression was found in the same dataset. This observation is, however, conflicting with some previous studies [22], but in line with others [21]. Our findings sustain the downregulation of *SIRT1* in PCa, as well as in PIN lesions, supporting the hypothesis that sirtuin 1 is involved in *H2AFZ* regulation. It should be recalled that *SIRT1* has been considered an oncogene and a tumor suppressor, depending on the cancer model under consideration [17, 29]. Indeed, it is known that sirtuin 1 is responsible for acquisition of genetic mutations in *BCR-ABL* in chronic myelogenous leukemia cells, conferring resistance to imatinib mesylate, which inhibits that tyrosine kinase [30, 31]. Additionally, *SIRT1* inhibits p53 through deacetylation, thus increasing the possibility of neoplastic transformation [32], p53 regulates *SIRT1* transcription through a negative feedback loop, decreasing *SIRT1* activity, which, in turn, increases *TP53* activity [33]. Moreover, *SIRT1* has been suggested as a tumor suppressor in colon cancer due to its ability to deacetylate and inactivate oncogenic β- catenin [23]. In the same vein, BRCA1 enhances sirtuin 1 activity, reducing cell growth and increasing apoptosis in breast cancer cells [25]. In PCa, sirtuin 1 has been involved in autophagy, and *SIRT1* knockdown induced PIN development, suggesting that *SIRT1* might be a tumor suppressor gene in this cancer model [24].

We found that *SIRT1* induced overexpression in PCa cell lines decreased H2A.Z protein levels. Since sirtuin 1 inhibits the mTOR pathway [26], we speculated whether H2A.Z downregulation due to sirtuin 1 overexpression might be mediated by repression of mTOR pathway, which is overactive and associated with tumor progression in PCa [34, 35]. Our findings suggest that, although mTOR inhibition might contribute to sirtuin 1-related downregulation of H2A.Z, it is not the main responsible as *FRAP* stably silenced PCa cell lines showed only a slight decrease in H2A.Z levels. Thus, other mechanisms are involved in regulation of H2A.Z by sirtuin 1. In fact, when simultaneous *SIRT1* overexpression and exposure to bortezomib were performed, levels of H2A.Z modestly decreased, depicting that proteasome has a major importance in sirtuin 1-mediated H2A.Z degradation, as previously described [16].

Because sirtuin 1 is a Class III HDAC, we hypothesized that post-translational histone modification might be involved in *H2AFZ* deregulation in PCa. Remarkably, exposure to epigenetic drugs (TSA alone or in combination with 5-aza-dC) resulted in increased *H2AFZ* levels in PCa cells and these results were corroborated by ChIP, which demonstrated an enrichment of histone activating marks, such as AcH3 and H3K4me2, in the TSS proximity. Nonetheless, H2A and H2A.Z levels remained stable after drug exposure and the deposition pattern of both histones suggest that H2A.Z, and especially acH2A.Z, replace H2A in nucleosomes next to the TSS, which makes the gene prone to be actively transcribed. Interestingly, this pattern is characteristic of oncogenes and actively transcribed genes [15, 36, 37], and supports an oncogenic function for *H2AFZ*. Nevertheless, and despite increased gene transcription, protein levels of H2A.Z decreased after exposure to TSA, indicating that a post-translational mechanism was involved in regulation of H2A.Z levels. Based on our results, we postulate that sirtuin 1 is responsible for that regulation. Indeed, we found that *SIRT1* expression was increased, both at transcript and protein levels, in all PCa cell lines after treatment with TSA. Moreover, enrichment in activating marks – specifically acH3 and H3K4me2 – was observed near the TSS of *SIRT1* gene, with a simultaneous decrease of the repressive mark H3K27me3. Hence, our results strongly suggest that epigenetic modulating drugs are able to increase *SIRT1* expression through histone post translational modifications. Remarkably, in contrast to *H2AFZ*, although levels of H2A.Z were increased nearby the *SIRT1* TSS, the canonical histone was not depleted, depicting a common feature of poised or poorly transcribed genes in cancer cells [15]. Importantly, we demonstrated that H2A.Z is not acetylated within nucleosomes neighboring *SIRT1* TSS, either before or after TSA exposure, which is in accordance with a previous study in PCa, regarding TSG [15]. Interestingly, it has been reported that sirtuin 1 not only regulates H2A.Z levels, but also that H2A.Z itself might influence *SIRT1* expression, suggesting a reciprocal regulation mechanism between both proteins [7,16]. Thus, we are tempted to speculate that H2A.Z overexpression negatively regulates *SIRT1*, resulting in the lower sirtuin 1 levels observed in PCa cell lines and primary tissues.

To ascertain the impact of sirtuin 1 activity on H2A.Z levels, we tested an inhibitor (nicotinamide) and an activator (resveratrol) of sirtuin 1 alone or combined with epigenetic modulating drugs. Remarkably, the effect on H2A.Z protein levels was independent of exposure to the epigenetic modifying drugs, demonstrating that sirtuin 1 also regulates H2A.Z levels through its direct enzymatic activity. Intriguingly, we found that nicotinamide induced a decrease of sirtuin 1 levels and similar results have already been reported in leukemic cells [38]. Contrarily, resveratrol alone induced an increase in sirtuin 1 protein levels, which is line with previous reports showing that it promotes *SIRT1* expression [39]. Hence, the combined effect of activation and induced expression of sirtuin 1 by resveratrol substantiates the observed decrease in H2A.Z protein levels. Importantly, exposure to epigenetic modifying drugs augmented this effect, mainly through increased *SIRT1* expression, as expected.

Importantly, we found that the mechanism by which sirtuin 1 promotes H2A.Z downregulation involves proteasomal-mediated degradation. This mechanism has been previously demonstrated in cardiomyocytes, in which increased deacetylation of Lys-15 triggers H2A.Z ubiquitylation on Lys-115 and Lys-121 and, ultimately, its degradation by the proteasomal pathway [16]. To further support this hypothesis in PCa cells, we showed, through PLA, that exposure to epigenetic modulating drugs dramatically increased the interaction between sirtuin 1 and H2A.Z, promoting their physical contact which is critical for degradation.

Interestingly, H2A.Z, a histone variant consistently associated with the malignant phenotype, might constitute an interesting therapeutic target [27]. Concerning the mechanism that regulates H2A.Z incorporation into nucleosomes, the ATP dependent nucleosome remodeler SNF-2-related CREB-binding protein activator protein (SRCAP) inserts H2A.Z into nucleosomes and deposits it near gene promoters [40, 41]. Remarkably, SRCAP has been already reported to be overexpressed in PCa [42]. Additionally, p400/Tip60, another complex responsible for H2A.Z insertion into chromatin, is also overactive in PCa [43]. Although a therapy based on SRCAP or p400/Tip60 repression would seem attractive, specific targeting of those complexes might entail high toxicity to non-neoplastic cells. Furthermore, it has been reported that H2A.Z and c-Myc, a transcription factor highly expressed in PCa [44], positively regulate each other, with high levels of the histone variant colocalized in *C-MYC* promoter during active transcription [9,12]. Hence, targeting of c-Myc might not only regulate this protein, but it would also allow for indirect regulation of H2A.Z. Nevertheless, anti-c-Myc based therapy is changeling and displays high toxicity, since *C-MYC* expression is ubiquitous in all proliferating, including non-malignant, cells [45].

Interestingly, a close correlation between sirtuin 1 and c-Myc has been already reported, and a synergistic effect has been demonstrate for these two proteins [46-49], suggesting an oncogenic role for sirtuin 1. However, it is known that the oncogenic role of sirtuin 1 is tumor- and context-specific, and the opposite relation between the histone deacetylase and c-Myc has already been described, indicating that sirtuin 1 promotes c-Myc instability [50]. Although in a different mechanistic context, we also observed a negative correlation between sirtuin 1 and c-Myc oncoprotein. Hence, we demonstrated that epigenetic modulation of H2A.Z, mediated by sirtuin 1 upregulation, decreased c-Myc expression, which might provide a more effective and less toxic alternative.

**Figure 7:**
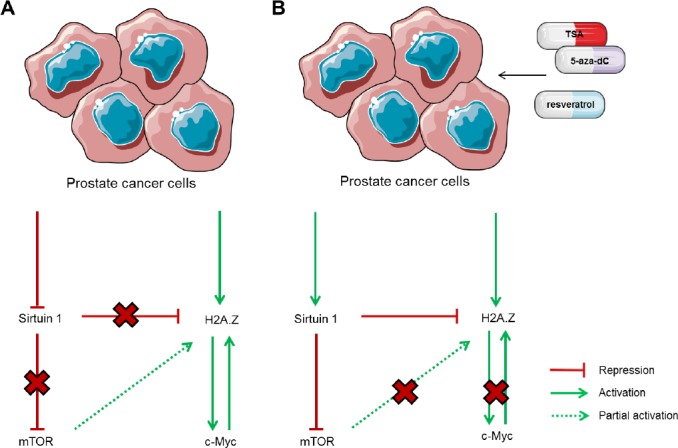
Interplay among sirtuin 1, H2A.Z, mTOR and c-Myc in prostate cancer cells (A) Under normal conditions, PCa cells display downregulation of SIRT1, thus, sirtuin 1 is unable to promote H2A.Z negative regulation directly, through its degradation, and indirectly, through mTOR pathway repression, leading to upregulation of c-Myc and other oncogenes. (B) When exposed to TSA, alone or combined with 5-aza-dC, or resveratrol, levels of sirtuin 1 are restored, leading to decreased levels of H2A.Z, partially due to mTOR pathway inhibition, but mostly via direct interaction between sirtuin 1 and the histone variant. Similarly, c-Myc levels decrease after treatment with those drugs.

In conclusion, we showed that sirtuin 1 and H2A.Z deregulation in PCa are reciprocally related. Epigenetic mechanisms, mostly histone post-translational modifications, are likely involved and impair sirtuin 1-mediated downregulation of H2A.Z *via* proteasome-mediated degradation. Epigenetic modifying drugs in conjunction with enzymatic modulators are able to restore the normal functions of sirtuin 1 and might constitute relevant tools for targeted therapy of PCa patients (Fig. 7).

## MATERIALS AND METHODS

### Patients and Samples

Fifty-seven PCa samples were prospectively collected from patients diagnosed and treated with radical prostatectomy at the Portuguese Oncology Institute – Porto, Portugal. From 10 of those patients, samples of PIN were also collected. Ten NPT, used as controls, were obtained from the peripheral zone of prostates that did not harbor PCa of patients submitted to radical cystoprostatectomy for bladder cancer. After surgery, all tissues specimens were promptly frozen at -80ºC and subsequently cut in a cryostat for nucleic acid extraction.

From each specimen, fragments were routinely collected, formalin-fixed and paraffin-embedded for histopathological examination. An expert pathologist evaluated Gleason score and pathological staging. Relevant clinical data was collected from the clinical charts. This study was approved by the institutional review board (Comissão de Ética para a Saúde) of Portuguese Oncology Institute - Porto, Portugal.

### Prostate cancer cells

LNCaP cells were grown in RPMI 1640, DU145 cells were maintained in MEM and PC-3 cells were grown in 50% RPMI-50% F-12 medium (GIBCO), as recommended. All culture media were supplemented with 10% fetal bovine serum and 1% penicillin/streptomycin (GIBCO). Cells were maintained in an incubator at 37ºC with 5% CO_2_. All PCa cell lines were karyotyped by G-banding (for validation purposes) and routinely tested for *Mycoplasma spp*. contamination (PCR Mycoplasma Detection Set, Clontech Laboratories).

### Transient transfection

PCa cells were transiently transfected with transfection-ready DNA containing *SIRT1* transcript variant 1 (Origene Technologies), following manufacturer's instructions. After transfection, *SIRT1*-overexpressing cells were also exposed to 0.1μM bortezomib (Cell Signaling) and subsequently harvested for RNA and protein extraction.

### Short hairpin RNA (shRNA) and lentiviral transduction

Cells were transfected with mTOR or negative control shRNA using shRNA Lentiviral Particles (sc-35409-V and sc-108080, respectively, Santa Cruz Biotechnology), according to manufacturer's indications. Clones successfully silenced were selected using the recommended complete medium supplemented with 2-5 μg/mL puromycin, as recommended by manufacturer. Cells were then collected for RNA and protein extraction.

### Cell culture and treatment with epigenetic modulating drugs, nicotinamide and resveratrol

PCa cells treated with 1μM 5-aza-dC (Sigma) for 72h and 0.5μM TSA (Sigma), added the last 24h. Cells were also exposed to 20mM nicotinamide (Sigma) or 40μM resveratrol (Sigma) for 72h, both alone or in combination with TSA or 5-aza-dC and TSA, according to the previous regimen. For control purposes, the same cell lines were cultured without treatment for 72 hours (mock cells). For each condition, three biological replicates were performed. Both medium and drugs were replaced every 24h.

### RNA isolation and quantitative reverse-transcription PCR (qRT-PCR)

Total RNA from tissue samples and cell lines was isolated using Trizol® Reagent (Invitrogen) and all genomic DNA present in the samples was eliminated using TURBO DNA-free (Ambion, Applied Biosystems), following manufacturer's instructions. For each sample, first strand synthesis was performed using the High-Capacity cDNA Reverse Transcription Kit (Applied Biosystems). Expression of target genes was quantified using TaqMan expression assays [*H2AFZ* (Hs01888362_g1), *SIRT1* (Hs01009005_m1), *FRAP* (Hs00234508_m1)], acquired as pre-developed assays from Applied Biosystems and normalized to the expression of the endogenous control *GUSB* (Hs99999908_m1). The standard curve method was used to determine RNA levels. All samples were analysed in triplicate, and the mean value was used for data analysis.

### Whole-cell extracts and immunoblot

Whole-cell protein extraction was performed using complete RIPA Buffer (Santa Cruz Biotechnology Inc.). Briefly, 20μg of total protein were separated by SDS-PAGE, transferred to nitrocellulose membranes and probed with antibodies against H2A.Z (Cell Signaling), sirtuin 1 (Abcam), mTOR (Cell Signaling), S6 (Cell Signaling), pS6 (Cell Signaling), c-Myc (Abcam) or the endogenous control β-actin (Sigma). Finally, blots were developed using Immun-Star WesternC Kit, according to manufacturer's indications (BioRad). All the experiments were performed in triplicate. Relative optical density determination was performed using QuantityOne Software version 4.6.6. (Biorad) and normalized for the loading control, β-actin.

### Chromatin immunoprecipitation (ChIP) Assay

EZ-Magna ChIP G-Chromatin Immunoprecipitation Kit and the Magna Grip Rack (Millipore) were used to perform ChIP assay according to the manufacturer's instructions. For each chromatin immunoprecipitation assay, anti-H2A (Abcam), anti-H2A.Z (Abcam), anti-AcH2A.Z (Abcam), anti-H3 (Abcam), anti-AcH3 (Millipore), anti-H3K4me2 (Abcam), anti-Histone H3K27me3 (Millipore) and the negative control provided with the kit (normal mouse IgG), were used.

DNA quantification was performed in a 7500 Real-Time PCR System (Applied Biosystems), using Power SYBR Green PCR Master Mix (Applied Biosystems). Three gene-specific pairs of primers for each gene promoter were used, in which primers A were located closer to TSS and C those that were more distant upstream TSS (Supp. Table 1). The relative amount of promoter DNA was normalized using Input Percent Method.

### Proximity ligation assay (PLA)

PC-3 cells, cultured in 1cm^2^ coverslips, were exposed to 0.5μM TSA (Sigma), alone or combined with 1μM 5-aza-dC (Sigma), and to 40μM resveratrol (Sigma), as previously mentioned. Additionally, Mock cells were used as controls. At the end of treatment, cells were fixed in 4% formaldehyde (Sigma) for 10 minutes and permeabilized in 0.5% Triton X-100 (Sigma), for 5 minutes, at room temperature and gently stirred.

PLA assay was performed using the commercial kit Duolink In Situ (OLINK Bioscience), according to manufacturer's instructions. The antibodies used were anti-sirtuin 1 (Abcam) and anti-H2A.Z (Cell Signaling). After the procedure, slides were evaluated under a fluorescence microscope (Olympus IX51, Olympus). PLA signals were counted in fifty randomly selected cells for each treatment.

### Statistical analysis

In cell lines, differences in transcript and protein levels among the treatments performed were determined using One-Way Analysis of Variance (one-Way ANOVA), followed by a multiple comparison Dunnet's (post-hoc) test, or Student's t-test, as appropriate, comparing all groups against the Mock.

Differences in quantitative expression levels of *SIRT1* and *H2AFZ* among NPT, PIN and PCA were assessed by the nonparametric Kruskall-Wallis test, followed by pairwise comparisons through Mann-Whitney *U*-test with Bonferroni's correction, when appropriate. The relationship between expression levels and standard clinicopathological variables (Gleason score, pathological stage) were assessed using the Kruskall- Wallis or the Mann-Whitney tests, as appropriate.

All tests were two-sided and statistical significance was set at *p*<0.05. Statistical analysis was performed using GraphPad Prism version 5.0 for Windows (GraphPad Software).

## Supplementary Figures and Table




