# Supplementary material for: Regulation of histone H2A.Z expression is mediated by sirtuin 1 in prostate cancer
Source: Oncotarget. 2013 Aug 29;4(10):1673–85. doi: 10.18632/oncotarget.1237 (PMC3858554; doi:10.18632/oncotarget.1237)
Supplement: Supplementary file 1 [file oncotarget-04-1673-s001.pdf]

Regulation of histone H2A.Z expression is mediated by sirtuin 1 in prostate cancer – Baptista et al

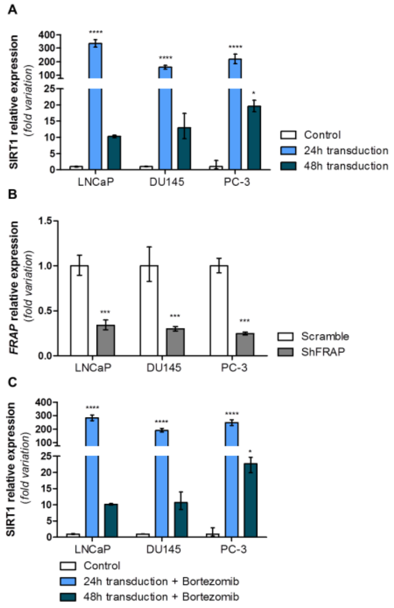

Supplementary Figure 1: Validation of effective (A) and (C) *SIRT1* overexpression and (B) *FRAP* silencing.

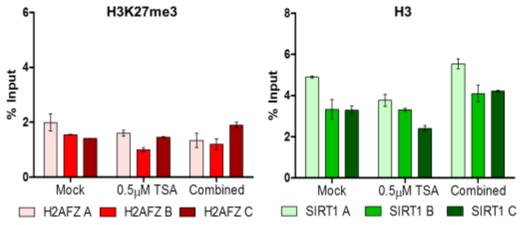

Supplementary Figure 2: ChIP assay results in LNCaP cell line regarding H3K27me3 and H3 across *H2AFZ* and *SIRT1* promoters, respectively. Results are normalized with the input of total sonicated chromatin (mean±SD).

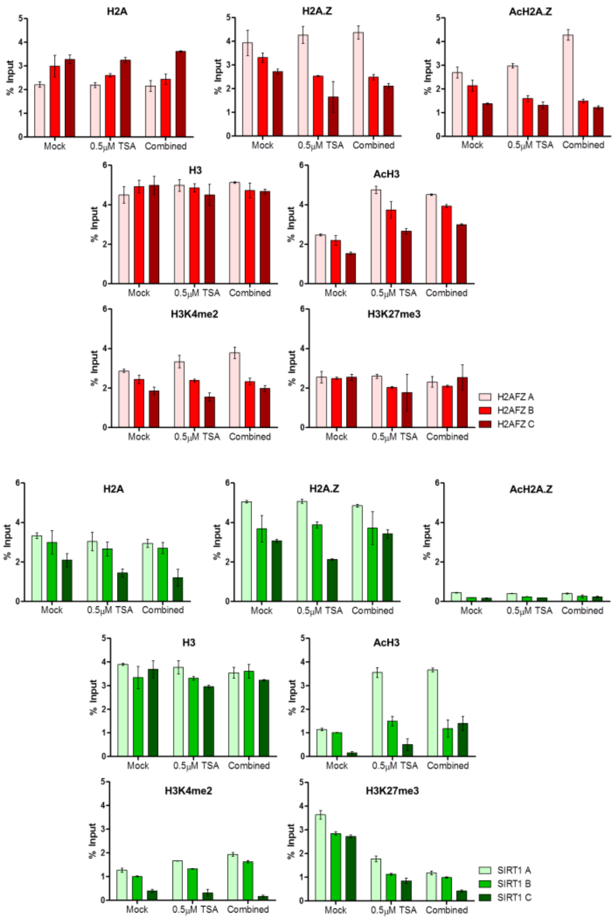

Supplementary Figure 3: ChIP assay results in DU145 cell line regarding H2A, H2A.Z, AcH2A.Z, H3, AcH3 H3K4me2 and H3K27me3 histones and histones marks across *H2AFZ* promoter and concerning the same histones and histones marks along *SIRT1* promoter. Results are normalized with the input of total sonicated chromatin (mean±SD).

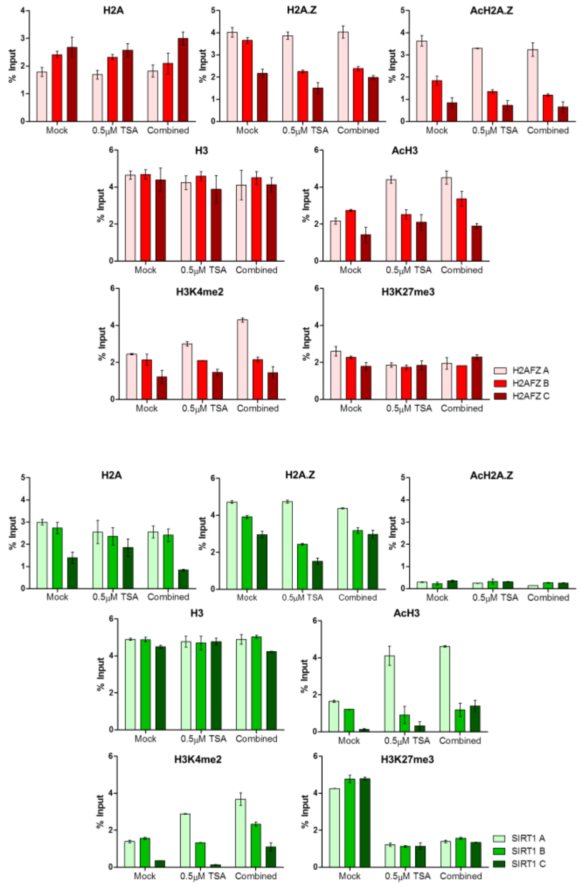

Supplementary Figure 4: ChIP assay results in PC-3 cell line regarding H2A, H2A.Z, AcH2A.Z, H3, AcH3 H3K4me2 and H3K27me3 histones and histones marks across *H2AFZ* promoter and concerning the same histones and histones marks along *SIRT1* promoter. Results are normalized with the input of total sonicated chromatin (mean±SD).
