# Supplementary material for: Regulation of histone H2A.Z expression is mediated by sirtuin 1 in prostate cancer
Source: Oncotarget. 2013 Aug 29;4(10):1673–85. doi: 10.18632/oncotarget.1237 (PMC3858554; doi:10.18632/oncotarget.1237)
Supplement: Supplementary file 2 [file oncotarget-04-1673-s002.pdf]

Regulation of histone H2A.Z expression is mediated by sirtuin 1 in prostate cancer – Baptista et al

Supplementary Table 1 - Features of primers used on ChIP assays

Gene		Primer	Sequence	Distance from TSS	$T_{ANNEALING}$ (ºC)
H2AFZ	A	Forward<br>Reverse	5' agcgtagctcgctctgtttc 3'<br>5' atgagcaagcgaagaaaagg 3'	-228bp	60
	B	Forward<br>Reverse	5' gtgacttggcttcaccgtt 3'<br>5' ttcttccgtctaggcgagtc 3'	-733bp	60
	C	Forward<br>Reverse	5' agacactactgtccccaagca 3'<br>5' gtccatgcaatgcaatcagt 3'	-1140bp	60
SIRT1	A	Forward<br>Reverse	5' tacacttcaggaagacgtggaa 3'<br>5' ccgctttctcaacttctcttc 3'	-310bp	62
	B	Forward<br>Reverse	5' tgcacgtgagaaaactgagg 3'<br>5' acctttgacgtggaggtttg 3'	-714bp	62
	C	Forward<br>Reverse	5' tatggccagaacccatactagg 3'<br>5' ggacccatataacccatggtag 3'	-1127bp	60
